# Analysis of factors associated with death in critically ill patients with COVID-19

**DOI:** 10.1590/0034-7167-2025-0378

**Published:** 2026-10-19

**Authors:** Maximina de Barros Cunha, Dulce Aparecida Barbosa, Cassiane Dezoti da Fonseca, Matheus Santos Moitinho, Julia Yaeko Kawagoe

**Affiliations:** I Universidade Federal de São Paulo. São Paulo, São Paulo, Brazil; II Universidade Federal de Sergipe. São Cristóvão, Sergipe, Brazil; III Faculdade Israelita de Ciências da Saúde Albert Einstein. São Paulo, São Paulo, Brazil

**Keywords:** COVID-19, Survival, Death, Intensive Care Units, Risk Factors., COVID-19, Sobrevivientes, Muerte, Unidades de Cuidados Intensivos, Factores de Riesgo.

## Abstract

**Objectives:**

to assess the factors associated with death in critically ill COVID-19 patients.

**Methods:**

retrospective cohort of critically ill patients admitted to the Intensive Care Unit and infected with SARS-CoV-2, followed for 30 days, ≥ 18 years old, with a positive nasopharyngeal RT-PCR test for SARS-CoV-2. Descriptive analysis regarding the outcome, chi-square test, and backward stepwise multiple logistic regression model were used to describe predictor variables of sociodemographic and clinical characteristics.

**Results:**

715 patients were analyzed, of whom 70.8% (n=507) died and 29.1% (n=209) survived. In the multivariate analysis, death outcome dependent variables, such as age range of 61-80 years, > 80 years, invasive mechanical ventilation, dialysis, corticosteroids, vancomycin, platelets after 24 hours, and other antibiotics, were identified as risk factors, and the ROC curve (AUC=0.904) predicted the proposed model.

**Conclusions:**

the multivariate model showed good discriminatory capacity, identifying independent factors associated with death in critically ill COVID-19 patients.

## RESUMEN

**Objetivos:**

evaluar los factores asociados con la muerte en pacientes gravemente enfermos con COVID-19.

**Métodos:**

cohorte retrospectiva de pacientes gravemente enfermos ingresados en la Unidad de Cuidados Intensivos e infectados con SARS-CoV-2, seguidos durante 30 días, ≥ 18 años, con una prueba RT-PCR nasofaríngea positiva para SARS-CoV-2. Se utilizó un análisis descriptivo del resultado, la prueba de chi-cuadrado y un modelo de regresión logística múltiple por pasos hacia atrás para describir las variables predictoras de las características sociodemográficas y clínicas.

**Resultados:**

se analizaron 715 pacientes, de los cuales el 70,8% (n=507) falleció y el 29,1% (n=209) sobrevivió. En el análisis multivariado, las variables dependientes del resultado de muerte, como el rango de edad de 61 a 80 años, > 80 años, ventilación mecánica invasiva, diálisis, corticosteroides, vancomicina, plaquetas después de 24 horas y otros antibióticos, se identificaron como factores de riesgo, y la curva ROC (AUC=0,904) predijo el modelo propuesto.

**Conclusiones:**

el modelo multivariado mostró una buena capacidad discriminatoria, identificando factores independientes asociados con la muerte en pacientes con COVID-19 grave.

## INTRODUCTION

Since its emergence in December 2019, Severe Acute Respiratory Syndrome coronavirus 2 (SARS-CoV-2) has posed an unprecedented challenge to healthcare systems worldwide, at various levels of the healthcare system, particularly in Intensive Care Units (ICUs)^(1)^. Considering the metrics of the devastation caused by the COVID-19 pandemic worldwide, Brazil ranked second among countries with the highest number of deaths from COVID-19 during 2021^(2)^. Based on notifications from the Influenza Epidemiological Surveillance Information System (SIVEP-Gripe - *Sistema de Informação e Vigilância Epidemiológico da Gripe*), ICU admissions totaled 245,304 (36.17%) cases, corresponding to 36.17% of the total. Of these, 115,018 (46.89%) were ICU admissions, resulting in 64,589 (56.16%) deaths. During the second wave, 130,286 (53.11%) cases were admitted to the ICU, with 83,781 (64.31%) deaths^(2,3)^.

In Brazil, during 2021, more than 70% of hospitalizations were covered by the Brazilian Unified Health System, primarily serving the most vulnerable population groups^(4)^. Given these alarming epidemiological data, efforts and strategies to control the acute phase of the disease were intensified, and only with the start of vaccination, which began in January 2021 in Brazil, covering older adults, was the beginning of a decline in the number of deaths observed^(5)^.

Few studies have investigated mortality in priority groups. Furthermore, this topic is challenging for the Brazilian healthcare system, especially when studying the variability of mortality data due to socioeconomic and health disparities in Brazil^(6)^, analyzing the epidemiological variables that may influence the context of critically ill patients admitted to the ICU. In Brazil, however, mortality rates varied alarmingly, ranging from 24.5% to 97.2% in different regions of the country^(7)^. In 2020, the majority of deaths occurred in older adults who commonly presented with multiple comorbidities, consequently increasing the difficulty in identifying the cause of death. Despite the high mortality rates, there was significant underreporting of deaths caused by COVID-19 due to diagnostic uncertainty and because it was an emerging disease^(8)^.

International studies comparing survivors and patients who died indicate that sex, comorbidities, laboratory abnormalities, inflammatory biomarkers, and the use of supportive therapies, such as mechanical ventilation and dialysis, are associated with mortality from COVID-19^(9-11)^. In Brazil, this data is heterogeneous and underexplored, reinforcing the need to identify clinical risk factors to guide targeted interventions^(12)^. Therefore, understanding which groups of individuals are most vulnerable to more severe manifestations, for the purpose of predicting death, from the perspective of surviving patients and those who died, will allow us to describe the epidemiology of patients admitted to Brazilian ICUs for critical care of COVID-19 patients.

## OBJECTIVES

To assess the factors associated with death in critically ill COVID-19 patients.

## METHODS

### Ethical aspects

The study was previously approved by the *Universidade Federal de São Paulo* Research Ethics Committee, with exemption from the requirement of the Informed Consent Form, since secondary data were analyzed and given the justifiable impossibility of obtaining participants’ consent.

### Study design, setting, and period

The single-center retrospective cohort study followed STrengthening in Reporting of OBservational studies in Epidemiology recommendations^(13)^. This study was conducted at a university hospital located in the city of São Paulo, Brazil. During the pandemic, the hospital reorganized its structure, flow, and hospital care to meet the growing demand for critically ill patients, making ICU beds available in various sectors for the exclusive care of patients affected by severe COVID-19. The data collection period was from March 2020 to April 2022.

### Population, inclusion and exclusion criteria

Adult patients (≥ 18 years) of both sexes, admitted to the ICU with a clinical picture classified as severe, defined by the need for intensive support such as invasive or non-invasive mechanical ventilation (IMV), use of vasoactive drugs and/or presence of acute respiratory failure, hemodynamic instability or organ dysfunction, were included^(14)^. The diagnosis of SARS-CoV-2 infection was confirmed by real-time reverse transcription polymerase chain reaction (RT-PCR) testing on a nasopharyngeal swab sample. Only patients with complete records of their clinical course in their institutional electronic medical records were included. Pregnant women, patients in palliative care, and cases with incomplete or inconsistent data were excluded.

### Study protocol

Data collection from medical records was performed retrospectively and longitudinally using a standardized and structured form developed on the Research Electronic Data Capture platform. An instrument was created to track records over time. The demographic and clinical data form was completed only once, and the results of laboratory tests were recorded upon admission, at 24 hours, at 48 hours, and on the seventh day of hospitalization. Laboratory results included hemoglobin, leukocytes, platelets, creatinine, urea, and D-dimer. The primary outcome was death, while the secondary outcome included the use of supportive therapies such as mechanical ventilation and dialysis. Follow-up lasted 30 days, considering ICU admission as the baseline. Factors associated (independent variables) with the primary outcome were sociodemographic data, clinical characteristics, and laboratory tests. Demographic and clinical variables included age, sex, comorbidities (hypertension, arrhythmias, diabetes mellitus, cardiovascular diseases, respiratory diseases, HIV, cancer, liver diseases, obesity, kidney transplantation, dyslipidemia, alcoholism, neurological diseases, and smoking), and medication use in the ICU.

### Analysis of results and statistics

For data analysis, quantitative variables were assessed for normality and homogeneity using the Shapiro-Wilk test and Levene’s test, respectively. These variables were presented as mean and standard deviation (±) when within normality, or as median and interquartile range (IQR). The sample size was determined by the number of eligible patients during the study period. No prior sample size calculation was performed as it was a convenience sample. Categorical variables were presented using absolute and relative frequencies (%). The sample was divided into two groups based on the survivor and non-survivor outcome. For continuity variables, the Mann-Whitney U test was used. For the analysis of associations between categorical variables, Pearson’s chi-square test (χ2) was used, with correction by Yates’ method as needed. Logistic regression with a stepwise backward method and comparison by the Likelihood Ratio test was conducted to identify independent variables associated with the death outcome. All independent variables with p<0.2 were imputed to the initial model, performing six stages. This automated method progressively removed the least significant variables until the final model was obtained. To adjust for multicollinearity, a chi-square matrix was performed with all independent variables of the proposed final model.

The model estimators were assessed as *Odds Ratio* (OR). Akaike’s information criterion was used as a comparative metric for the quality of the generated statistical models. For all analyses, a 95% Confidence Interval (95%CI) was adopted, with a p-value <0.05 considered statistically significant. For significant analyses, the biserial correlation coefficient and Cramer’s V were used to assess the magnitude of the effect size of the results. RStudio^®^ 2023.12.0 and Jamovi^®^ 2.2.5 were used for all analyses.

## RESULTS

We analyzed 715 patients with COVID-19, of whom 506 (70.7%) died in the hospital (non-survivors) and 209 (29.2%) were discharged (survivors). Table 1 presents the data. The median age was 64 years (IQR=55-73), which was related to the death outcome (p<0.01). Notably, non-survivors were, on average, nine years older than survivors. The percentage of male patients was higher in the survivor group (65.4%).

**Table 1:** Demographic and baseline characteristics of critically ill COVID-19 patients in an Intensive Care Unit, São Paulo, São Paulo, Brazil, 2025

Demographic and baseline characteristics	Total (N=715)	Outcome		Statistical test *p* value
		Survivors	Non-survivors	
		(n=209)	(n=506)	
**Male n(%)**	450 (64.0)	136(65.4)	314 (63.4)	χ^2^_1_=0.24. p=0.62^2^
**Age Md (IQR)**	64 (55-73)	57 (47.7.67)	66 (58.75)	**U=34274. p<0.01^1^**
**Race n(%)**				χ^2^_3_=0.65. p=0.88^2^
White	297 (59.2)	120 (58.5)	417 (59.0)	
Black	60 (12.0)	27 (13.2)	87 (12.3)	
Mixed-race	144 (28.7)	57 (27.8)	201 (28.4)	
Indigenous	1 (0.2)	1 (0.5)	2 (0.3)	
**Comorbidities n(%)**	669 (94.2)	189 (91.3)	480 (95.4)	**χ^2^_1_ =4.58. P=0.03^2^**
Hypertension	453 (67.9)	126 (66.7)	327 (68.4)	χ^2^_1_=0.19. p<0.66^2^
Arrhythmias	41 (6.2)	8 (4.3)	33 (6.9)	χ^2^_1_=1.67. p<0.20^2^
Diabetes mellitus	272 (40.9)	80 (42.6)	192 (40.3)	χ^2^_1_=0.30. p<0.59^2^
Cardiovascular disease	162 (24.4)	24 (12.7)	138 (29.1)	**χ^2^_1_ =19.60. p<0.01^2^**
Chronic obstructive pulmonary disease and/or asthma	85 (12.7)	21 (11.2)	64 (13.4)	χ^2^_1_=0.58. p<0.45^2^
HIV/AIDS	17 (2.5)	4 (2.1)	13 (2.7)	χ^2^_1_=0.20. p<0.66^2^
Cancer	77 (11.5)	13 (6.9)	64 (13.4)	**χ^2^_1_ =5.62. p<0.02^2^**
Liver diseases	36 (5.4)	2 (1.1)	34 (7.1)	**χ^2^_1_ =9.75. p<0.02^2^**
Obesity	104 (15.7)	41 (21.7)	63 (13.3)	**χ^2^_1_ =7.27. p<0.01^2^**
Kidney diseases	147 (22.1)	33 (17.6)	114 (23.8)	χ^2^_1_=3.11. p<0.08^2^
Dyslipidemia	85 (12.8)	33 (17.5)	52 (10.9)	**χ^2^_1_ =5.23. p<0.02^2^**
Alcoholism	71 (10.7)	15 (8.0)	56 (11.7)	χ^2^_1_=1.92. p<0.17^2^
Neurological diseases	59 (8.8)	9 (4.8)	50 (10.5)	**χ^2^_1_ =5.45. P<0.02^2^**
Smoking	200 (30.0)	62 (32.8)	138 (28.9)	χ^2^_1_=1.00. p<0.32^2^
**Medications in the ICU n(%)**				
Oseltamivir	87 (12.4)	39 (19.4)	48 (9.6)	**χ^2^_1_ =12.60. p<0.01^2^**
Ceftriaxone	441 (62.3)	125 (61.0)	316 (62.8)	χ^2^_1_=0.21. p<0.65^2^
Clarithromycin	165 (23.4)	50 (24.5)	115 (23.0)	χ^2^_1_=0.20. p<0.66^2^
Azithromycin	300 (42.3)	108 (52.7)	192 (38.0)	χ^2^_1_=12.85. p<0.01^2^
Vancomycin	481 (67.7)	81 (39.5)	400 (79.2)	χ^2^_1_=105.15. p<0.01^2^
Piperacillin tazobactam	255 (36.0)	70 (34.3)	185 (36.6)	χ^2^_1_=0.34. p<0.56^2^
Hydroxychloroquine	126 (18.0)	55 (27.2)	71 (14.3)	**χ^2^_1_ =16.28. p<0.01^2^**
Corticosteroids	473 (68.3)	100 (50.3)	373 (75.5)	**χ^2^_1_ =41.76. p<0.01^2^**
Anticoagulants	631 (91.1)	191 (96.5)	440 (88.9)	**χ^2^_1_ =9.96. p<0.01^2^**
**Medications for chronic use**	642 (89.7)			**χ^2^_1_ =9.96. p<0.01^2^**
No	102(15.9)	44(21.9)	58(13.1)	
Yes	354(55.1)	135(67.2)	219(49.5)	
**Renal replacement therapy n(%)**	415 (58.2)	61 (29.3)	354 (70.1)	**χ^2^_1_ =100.67. p<0.01^2^**
**IMV n(%)**	574 (80.2)	123 (58.9)	451 (89.0)	**χ^2^_1_ =84.35. p<0.012^2^**

HIV - Human Immunodeficiency Virus; AIDS - Acquired Immunodeficiency Syndrome; ICU - Intensive Care Unit; IMV - invasive mechanical ventilation; IQR - Interquartile Range Md - median; 2χ - Pearson;

3 χ - Yates continuity correction.

Concerning race, there was no statistically significant difference between groups. Comorbidities were significant for the death outcome, especially cardiovascular diseases (29.1%), cancer (13.4%), obesity (13.3%), dyslipidemia (10.9%), neurological diseases (10.5%), and liver diseases (7.1%) (p<0.05) for all these analyses. Patients using chronic medications accounted for 55.1% of the sample, and of those who used them regularly and continuously, 67.2% were from the survivor group.

Patients undergoing mechanical ventilation and renal replacement therapy had high mortality rates of 70.1% and 89%, respectively (p<0.01). Regarding medications for treating in-hospital COVID-19 in ICUs, 49.5% of patients who were using them continuously did not survive (p<0.01).

Table 2 presents the laboratory tests and their association with death. Platelets, urea, and serum creatinine were elevated within 24 hours of ICU admission, with a further elevation on the seventh day after ICU admission (p<0.01).

**Table 2:** Abnormal laboratory test within 24 hours and on the seventh day of admission to the Intensive Care Unit, São Paulo, São Paulo, Brazil, 2025

Laboratory tests	Total (N=715)	Outcome		*p* value
		Survivors	Non-survivors	
		(n=209)	(n=506)	
C-reactive protein				
24 hours	528 (99.8)	150 (99.3)	378 (100.0)	χ^2^_1_=2.51. p=0.28^4^
Seventh day	567 (99.8)	167 (99.4)	400 (100.0)	χ^2^_1_=2.39. p=0.29^4^
**Hemoglobin**				
24 hours	364 (51.9)	105 (51.0)	259 (52.2)	χ^2^_1_=0.09. p=0.76^2^
Seventh day	41 (6.2)	8 (4.3)	33 (6.9)	χ^2^_1_=0.26. p=0.61^2^
**Hematocrit**				
24 hours	257 (57.4)	99 (55.0)	158 (59.0)	χ^2^_1_=0.69. p=0.41^2^
Seventh day	256 (56.3)	94 (52.2)	162 (58.9)	χ^2^_1_=1.98. p=0.16^2^
**Leukocytes**				
24 hours	290 (41.4)	78 (37.9)	212 (42.8)	χ^2^_1_=1.48. p=0.22^2^
Seventh day	433 (61.8)	117 (56.8)	433 (61.8)	χ^2^_1_=3.05. p=0.08^2^
**Platelets**				
24 hours	228 (32.5)	53 (25.7)	175 (35.3)	**χ^2^_1_=6.06. p=0.01^2^**
Seventh day	165 (23.5)	37 (18.0)	128 (25.8)	**χ^2^_1_=4.92. p=0.03^2^**
**Urea**				
24 hours	432 (62.2)	103 (50.2)	329 (67.1)	**χ^2^_1_=17.55. p<0.01^2^**
Seventh day	580 (83.2)	148 (72.2)	432 (87.8)	**χ^2^_1_=25.24. p<0.01^2^**
**Creatinine**				
24 hours	533 (76.8)	140 (68.0)	393 (80.5)	**χ^2^_1_=12.85. p<0.01^2^**
Seventh day	613 (87.9)	166 (80.6)	447 (91.0)	**χ^2^_1_=14.97. p=0.01^2^**
**D-dimer**				
24 hours	360 (95.5)	120 (94.5)	240 (96.0)	χ^2^_1_=0.45. p=0.50^2^
Seventh day	462 (97.7)	134 (96.4)	328 (98.2)	χ^2^_1_=1.40. p=0.24^2^

2 χ - Pearson;

4 Fisher’s exact test.

For the univariate logistic model, the results showed that age category, medication use in the ICU, comorbidities, renal replacement therapy, mechanical ventilation, and some laboratory findings were important factors associated with death.

Table 3 shows that, in relation to age, individuals in the 31-60 age group had 4.5 times greater odds of death (OR: 4.51; 95%CI: 1.68 - 12.12; p = 0.003) and those in the 61-80 age group had 6.3 times greater odds of death (OR: 6.30; 95%CI: 1.96 - 20.21; p = 0.002), both when compared to those in the 18-30 age group. Males did not show a significant association with death (OR: 0.91; 95%CI: 0.65 - 1.28; p = 0.623).

**Table 3:** Exponential distribution of estimated coefficients according to the death outcome, *Odds Ratio*, and 95% Confidence Interval, São Paulo, São Paulo, Brazil, 2025

Univariate predictor of death		*Odds Ratio*	95%CI	*p* value
**Age category (years)**	**18-30 years**	**1.50**	(0.56-4.01)	0.416
	**31-60 years**	**4.51**	**(1.68-12.12)**	**0.003**
	**61-80 years**	**6.30**	**(1.96-20.21)**	**0.002**
**Sex**	**Male**	**0.91**	(0.65-1.28)	0.623
**Medications in the ICU**	Oseltamivir	**0.44**	**(0.27-0.70)**	**<0.001**
	Ceftriaxone	1.08	(0.77-1.51)	0.646
	Clarithromycin	0.91	(0.62-1.34)	0.658
	Azithromycin	**1.81**	**(1.30-2.51)**	**<0.001**
	Vancomycin	**5.83**	**(4.09-8.30)**	**<0.001**
	Piperacillin tazobactam	1.10	(0.78-1.55)	0.56
	Hydroxychloroquine	**0.44**	**(2.99-0.66)**	**<0.001**
	Corticosteroids	3.05	(2.16-4.31)	**<0.001**
	Anticoagulants	**0.29**	**(0.13-0.65)**	**0.003**
**Comorbidities**		1.98	(1.04-3.76)	0.035
	Hypertension	1.08	(0.75-1.55)	0.664
	Arrhythmias	1.67	(0.75-3.69)	0.201
	Diabetes mellitus	0.90	(0.64-1.28)	0.587
	Cardiovascular diseases	2.81	(1.75-4.51)	**<0.001**
	Chronic obstructive pulmonary disease and/or asthma	1.22	(0.72-2.07)	0.446
	HIV/AIDS	1.29	(0.41-4.00)	0.659
	Cancer	**2.09**	**(1.12-3.89)**	**0.02**
	Liver diseases	**7.17**	**(1.70-30.17)**	**0.007**
	Obesity	**0.55**	**(0.35-0.85)**	**0.008**
	Kidney transplant	1.47	(0.95-2.26)	0.079
	Dyslipidemia	0.57	(0.36-0.92)	0.023
	Alcoholism	1.52	(0.83-2.76)	0.168
	Neurological diseases	**2.33**	**(1.12-4.85)**	**0.023**
	Smoking	0.83	(0.57-1.19)	0.318
**Renal replacement therapy**		**5.65**	**(3.96-8.05)**	**<0.001**
**IMV**		**5.61**	**(3.79-8.30)**	**<0.001**
**Laboratory findings**	Hemoglobin after 24 hours	1.05	(0.75-1.45)	0.301
	Hemoglobin on the seventh day	1.08	(0.78-1.50)	0.608
			
	Hematocrit after 24 hours	1.17	(0.80-1.72)	0.407
	Hematocrit on the seventh day	1.31	(0.89-1.91)	0.160
			
	Lymphocytes after 24 hours	1.22	(0.88-1.71)	0.224
	Lymphocytes on the seventh day	1.34	(0.96-1.87)	0.081
			
	Platelets after 24 hours	1.57	(1.09-2.26)	**0.014**
	Platelets on the seventh day	1.58	(1.05-2.38)	**0.027**
			
	Urea after 24 hours	2.02	(1.45-2.82)	**<0.001**
	Urea on the seventh day	2.77	(1.84-4.16)	**<0.001**
			
	Creatinine after 24 hours	1.95	(1.34-2.81)	**<0.001**
	Creatinine on the seventh day	2.44	(1.54-3.89)	**<0.001**
			
	D-dimer after 24 hours	1.40	(0.52-3.76)	0.506
	D-dimer on the seventh day	2.04	(0.61-6.79)	0.246

*HIV - Human Immunodeficiency Virus; AIDS - Acquired Immunodeficiency Syndrome; ICU - Intensive Care Unit; IMV - invasive mechanical ventilation; 95%CI - 95% Confidence Interval.*

In relation to comorbidities, the presence of any comorbidity increased the odds of death by 98% (OR: 1.98; 95%CI: 1.04 - 3.76; p = 0.035), with emphasis on cardiovascular diseases (OR: 2.81; 95%CI: 1.75 - 4.51; p < 0.001), cancer (OR: 2.09; 95%CI: 1.12 - 3.89; p = 0.02), liver diseases (OR: 7.17; 95%CI: 1.70-30.17; p=0.007), obesity (OR: 0.55; 95%CI: 0.35-0.85; p=0.008), and neurological diseases (OR=2.33; 95%CI: 1.12-4.85; p=0.023). Therefore, comorbidities such as cardiovascular diseases, cancer, liver diseases, obesity, dyslipidemia, and neurological diseases were the significant chronic conditions for the death outcome (p<0.01). It is worth noting that when a patient underwent supportive therapies, such as mechanical ventilation or renal replacement therapy, their odds of dying increased more than 5.6 times for both: OR: 5.61; 95%CI: 3.96-8.05; p<0.001 and OR: 5.65, 95%CI: 3.79-8.30; p<0.001, respectively.

On the other hand, according to the findings of renal replacement therapy, biomarkers of renal function, such as serum urea and creatinine, were predictors of death with more than twice the risk of death on the seventh day, and platelets at 24 hours and on the seventh day demonstrated relevance in this case series, reaching, after seven days (OR:1.58; 95%CI:1.05-2.38; p=0.027).

For the final stepwise backward regression model (Table 4), we obtained the selected variables with p less than 0.2 and performed six stages with the final model. Multivariate analyses were then performed. After adjustment, 18 variables were included in the final model, and the vancomycin + IMV interaction, when added to the model for control purposes, showed better data fit and a significant difference compared to the final model.

**Table 4:** Final model of stepwise backward multivariate regression with and without adjustment for interaction, *Odds Ratio*, and 95% Confidence Interval for factors associated with the death outcome, São Paulo, São Paulo, Brazil, 2025

Variables	Unadjusted model			Adjusted model		
	*Odds Ratio*	95%CI	*p* value	*Odds Ratio*	95%CI	*p* value
18-30 years old	0.033	(0.006-0.164)	<0.001	0.03	(0.006-0.158)	<0.001
31-60 years old	0.108	(0.034-0.299)	<0.001	0.09	(0.026-0.273)	<0.001
61-80 years old	0.382	(0.124-1.047)	0.076	0.35	(0.103-1.044)	0.078
IMV	5.965	(3.251-11.21)	<0.001	28.5	(9.502-103.7)	<0.001
Admission to the ICU	0.198	(0.028-0.828)	0.051	0.24	(0.034-1.010)	0.087
Vancomycin	3.705	(2.217-6.258)	<0.001	29.6	(8.459-122.1)	<0.001
Corticosteroids	1.889	(1.129-3.164)	0.015	1.83	(1.090-3.100)	0.022
Anticoagulants	0.170	(0.047-0.506)	0.003	0.16	(0.040-0.512)	0.005
Cardiovascular diseases	4.599	(2.344-9.521)	<0.001	4.82	(2.375-10.47)	<0.001
Cancer	2.560	(1.135-6.228)	0.030	2.31	(1.012-5.723)	0.056
Liver disease	10.19	(2.392-72.10)	0.005	10.11	(2.380-71.15)	0.005
Obesity	0.584	(0.315-1.087)	0.088	0.590	(0.319-1.101)	0.094
Dyslipidemia	0.580	(0.282-1.199)	0.139	0.578	(0.279-1.213)	0.142
Neurological diseases	2.474	(0.880-7.966	0.104	2.635	(0.892-9.098)	0.099
Renal replacement therapy	6.135	(3.637-10.59)	<0.001	6.278	(3.683-10.96)	<0.001
Platelets after 24 hours	1.963	(1.036-3.741)	0.039	1.927	(1.011-3.693)	0.046
Platelets on the seventh day	0.505	(0.239-1.041)	0.068	0.507	(0.235-1.062)	0.076
Urea after 24 hours	0.663	(0.398-1.105)	0.114	0.667	(0.397-1.121)	0.125
Ventilation ^*^Vancomycin	-	-	-	0.077	(0.017-0.303	<0.001

ICU - Intensive Care Unit; IMV - invasive mechanical ventilation; 95% Confidence Interval - 95% Confidence Interval; Ventilation^*^Vancomycin - Ventilation + Vancomycin Interaction.

## DISCUSSION

The study revealed a high and concerning mortality rate among critically ill patients. These findings reinforce the severity of COVID-19, especially in ICU settings. Data analysis revealed that advanced age and the presence of comorbidities were the main factors for fatal outcomes and the use of supportive therapies, such as dialysis and mechanical ventilation, while biomarkers of renal, hematological, and hepatic functions stood out as critical indicators of the severity of patients’ clinical condition.

The high mortality rate in our study was discrepant compared to previous studies, which revealed lower rates ranging from 21.7% to 47.3% of hospitalized patients in Brazil. However, these studies originated from SIVEP-Gripe, and we can consider that the variation in these rates occurred due to different availability of access to health technologies in different regions of the country^(6,15)^.

Advanced age was a determining factor in the high number of non-surviving patients in this investigation. When we categorized patients by age group, we observed that their odds of death increased compared to younger patients. Older adults present a low-grade pro-inflammatory state which, together with the peculiarities of immunosenescence and the immune aging process, can favor cytokine storm syndrome and an increase in serum inflammatory mediators^(16)^.

This study found no statistical significance in the relationship between race and mortality rates. On the other hand, the study by Baqui *et al*.^(17)^ found two distinct effects, with an increase in mortality in the North region (regional effect) and in the mixed-race population (ethnic effect). It is speculated that the ethnic effect may be related to access to healthcare; therefore, our analysis supports this justification, since the Southeast region concentrated the majority of Brazil’s physical, human, and technological healthcare resources during the pandemic.

The presence of comorbidities was significantly associated with death (p=0.032), with 91.3% of survivors also presenting some pre-existing condition. Comorbidities may predispose to a dysfunctional immune response, negatively impacting COVID-19 evolution^(18)^. Cardiovascular diseases, in particular, are strongly linked to disease severity, potentially resulting in myocarditis, acute coronary syndromes, or heart failure, with a mortality risk up to five times higher compared to patients without cardiovascular disease^(19)^. In this study, cardiovascular diseases increased the odds of death by 2.81 times, in agreement with findings by Almeida-Pititto^(20)^.

Liver diseases appear as a significant factor in the mortality group, with more than seven times the odds of a worse prognosis, supporting data showing that chronic liver disease presented significantly higher odds of developing a severe form of the disease^(21)^. A COVID-19-dependent pro-inflammatory profile and immune suppression promote the ideal microenvironment for immune initiation and evasion of malignant cells, tumor progression and metastasis, contributing to the progression of various types of cancer and mortality from SARS-CoV-2^(22)^.

In addition to these comorbidities, this Brazilian study also found a higher prevalence of neurological diseases when characterizing the patient profile. The care of COVID-19 patients in the ICU, especially those with stroke, is described as being associated with high mortality rates. Therefore, neurological comorbidities deserve greater attention when directing this population towards proactive viral screening^(23)^.

This data allowed for a review of the main pre-existing comorbidities and how the Brazilian public healthcare system should prioritize these groups and maintain vaccination campaigns, avoiding the gap that occurred in understanding the preventive value of the vaccine and the hesitancy in its use, since it protects not only vulnerable individuals but also the development of severe illnesses and deaths from COVID-19^(24)^. Upon admission, 80.5% of patients presented evidence of acute kidney injury, assessed by serum creatinine, a routine test in the ICU. Previous studies report normal values for creatinine, sodium, and potassium, highlighting the importance of longitudinal monitoring from admission.

Although the present study did not stratify the type of kidney injury, this elevation in creatinine has been widely reported as a predictor of morbidity and mortality. Therefore, the attention given to daily creatinine monitoring in clinical practice establishes creatinine as a useful biomarker to highlight the need for special attention to renal patients with COVID-19. Another significant marker was urea, supporting its role as a marker of the characteristics of the inflammatory storm and disease severity^(25)^. Furthermore, the initiation of therapy accounted for 70.1% of deaths, a value that is discrepant when compared to data from the United States, where 20.6% of patients required renal replacement therapy within 14 days of ICU stay. These significant figures may be related to the direct viral damage mechanism mediated by the co-expression and activity of angiotensin-converting enzyme 2, important mechanisms that lead to kidney injury in patients with more critical infections^(26)^.

Many studies are emerging regarding the association between IMV and patient outcomes, which may be multifactorial. In our study, mortality was 89%, which is particularly high, similar to a cohort that, at 28 days, presented an 81.1% mortality rate^(27)^. These data suggest the vulnerability of patients on mechanical ventilation to ventilator-induced lung injuries, raising factors that are still widely debated when considering intubation strategies. However, when we analyze the interaction of the covariate IMV and vancomycin after model adjustment, we could point to another discussion regarding the increased use of antibiotics associated with IMV use in critical cases and secondary infections, triggering the indiscriminate use of broad-spectrum antibiotics^(28)^.

### Study limitations

The study featured a retrospective, single-center design based on secondary data from medical records for clinical purposes. The study covered the initial phase of the pandemic, including the first and second waves of COVID-19, characterized by high incidence and hospitalization, without considering the impact of different variants or vaccination, which may limit the generalizability of results to later phases of the disease. Although the design is cohort, logistic regression was chosen to identify independent factors associated with death, adjusting for multiple predictors simultaneously. It is recognized that, due to the high frequency of the outcome, ORs may overestimate the magnitude of the associations compared to the relative risk, and this limitation should be considered in the interpretation of results. However, logistic regression remains adequate for the selection of independent variables and the construction of explanatory models.

### Contributions to nursing, health, or public policy

This study allowed us to associate and analyze the death outcome due to COVID-19 during the early stages of the pandemic and to meticulously characterize this population, demonstrating the importance of promoting prevention and control of risk factors in groups most vulnerable to severity. Furthermore, the study has important contributions, demonstrated in the logistic regression model for predicting mortality, which may be similar in other epidemiological situations.

## CONCLUSIONS

Advanced age and chronic comorbidities, such as cardiovascular disease, liver disease, cancer, dialysis, mechanical ventilation, and clinical contexts of severity and co-infections, have been shown to be associated factors in predicting mortality in critically ill COVID-19 patients. The multivariate model showed good discriminatory capacity, identifying independent factors associated with death in critically ill COVID-19 patients.

## Data Availability

The research data are available in a repository: https://doi.org/10.48331/SCIELODATA.EULYWL.
