# Supplementary material for: Analysis of factors associated with death in critically ill patients with COVID-19
Source: Rev Bras Enferm. 2026 Oct 19;79:e20250378. doi: 10.1590/0034-7167-2025-0378 (PMC13679581; doi:10.1590/0034-7167-2025-0378)
Supplement: Translation

# Translation: Análise dos fatores associados ao óbito de pacientes graves com COVID-19

**DOI:** 10.1590/0034-7167-2025-0378pt

**Authors:** Maximina de Barros Cunha, Dulce Aparecida Barbosa, Cassiane Dezoti da Fonseca, Matheus Santos Moitinho, Julia Yaeko Kawagoe

**Affiliations:** I Universidade Federal de São Paulo. São Paulo, São Paulo, Brasil; II Universidade Federal de Sergipe. São Cristóvão, Sergipe, Brasil; III Faculdade Israelita de Ciências da Saúde Albert Einstein. São Paulo, São Paulo, Brasil

**Keywords:** COVID-19, Sobreviventes, Óbito, Unidades de Terapia Intensiva, Fatores de Risco.

## Abstract

**Objetivos:**

avaliar os fatores associados ao óbito de pacientes graves com COVID-19.

**Métodos:**

coorte retrospectiva de pacientes graves internados em Unidade de Terapia Intensiva e infectados com SARS-CoV-2, seguidos durante 30 dias, ≥ 18 anos, com exame nasofaríngeo de RT-PCR para SARS-CoV-2 positivo. Análise descritiva em relação ao desfecho, teste qui-quadrado e modelo de regressão logística múltipla *backward stepwise* foram utilizados para descrever as variáveis preditoras das características sociodemográficas e clínicas.

**Resultados:**

analisaram-se 715 pacientes, dos quais 70,8% (n=507) morreram e 29,1% (n=209) sobreviveram. Na análise multivariada, as variáveis dependentes do desfecho óbito, como faixa etária de 61-80 anos, > 80 anos, ventilação mecânica invasiva, diálise, corticoides, vancomicina, plaquetas após 24 horas e outros antibióticos, foram identificadas como fator de risco, e a curva ROC (AUC=0,904) apresentou predição do modelo proposto.

**Conclusões:**

o modelo multivariado apresentou boa capacidade discriminatória, identificando fatores independentes associados ao óbito em pacientes graves com COVID-19.

## INTRODUÇÃO

Desde o seu surgimento, em dezembro de 2019, a Síndrome Respiratória Aguda Grave-coronavírus-2 (SARS-CoV-2) configurou-se como um desafio sem precedentes para todos os ministérios de saúde ao redor do mundo, nos mais diferentes níveis do sistema de atenção à saúde, com destaque para as Unidades de Terapia Intensiva (UTIs)^(1)^. Tendo em mente as métricas da devastação da pandemia de COVID-19 mundialmente, o Brasil deteve o segundo lugar entre os países com maior número de mortes pela COVID-19 durante o ano 2021^(2)^. Com base nas notificações do Sistema de Informação e Vigilância Epidemiológico da Gripe (SIVEP-Gripe), as admissões em UTI totalizaram 245.304 (36,17%) casos, correspondendo a 36,17% do total. Desses, 115.018 (46,89%) foram internações em UTI, resultando em 64.589 (56,16%) óbitos. Durante a segunda onda, 130.286 (53,11%) casos foram admitidos na UTI, com 83.781 (64,31%) mortes^(2,3)^.

No Brasil, no período de 2021, mais de 70% dos das internações foram cobertas pelo Sistema Único de Saúde, atendendo prioritariamente grupos populacionais mais vulneráveis^(4)^. Devido a esses dados epidemiológicos alarmantes, crescentes esforços e estratégias foram incentivados para controlar a fase aguda da doença, e somente com o início da vacinação, que se iniciou em janeiro de 2021 no Brasil, abrangendo os indivíduos com idade avançada, que se observou o início da queda do número de óbitos^(5)^.

Poucas evidências consideráveis a despeito da mortalidade em grupos prioritários foram realizadas. Além disso, essa temática é desafiadora para o sistema de saúde brasileiro, principalmente quando se estuda sobre a variabilidade de dados da mortalidade devido às disparidades socioeconômicas e de saúde no Brasil^(6)^, analisando-se as variáveis epidemiológicas que podem influenciar o contexto dos pacientes graves internados em UTI. No Brasil, entretanto, as taxas de mortalidade variaram de forma alarmante, oscilando entre 24,5% e 97,2% em diferentes regiões do país^(7)^. Em 2020, a maioria dos casos de mortes ocorreu em idosos que comumente apresentavam múltiplas comorbidades, aumentando consequentemente a dificuldade na identificação da causa da mortalidade. Apesar dos altos índices de mortalidade, houve muita subnotificação de mortes causadas pela COVID-19, devido à incerteza do diagnóstico e por se tratar de uma doença emergente^(8)^.

Estudos internacionais que comparam sobreviventes e pacientes que foram a óbito indicam que sexo, comorbidades, alterações laboratoriais, biomarcadores inflamatórios e uso de terapias de suporte, como ventilação mecânica e diálise, estão associados à mortalidade por COVID-19^(9-11)^. No Brasil, esses dados são heterogêneos e pouco explorados, reforçando a necessidade de identificar fatores clínicos de risco para orientar intervenções direcionadas^(12)^. Dessa forma, compreender quais grupos de indivíduos estão mais vulneráveis a manifestações mais graves, para predição ao óbito, sob a ótica dos pacientes sobreviventes e dos que foram a óbito, permitirá descrever a epidemiologia dos pacientes admitidos nas UTIs brasileiras no atendimento crítico dos pacientes com COVID-19.

## OBJETIVOS

Avaliar os fatores associados ao óbito de pacientes graves com COVID-19.

## MÉTODOS

### Aspectos éticos

O estudo foi previamente aprovado pelo Comitê de Ética em Pesquisa da Universidade Federal de São Paulo, com isenção da exigência do Termo de Consentimento Livre e Esclarecido, uma vez que foram analisados dados secundários e diante da impossibilidade justificável de obtenção da anuência do participante.

### Desenho, local do estudo e período

O estudo de coorte retrospectivo em um único centro seguiu as recomendações do *STrengthening in Reporting of OBservational studies in Epidemiology* ^(13)^. Foi realizado em um hospital universitário localizado na cidade de São Paulo, Brasil. Durante a pandemia, o hospital reorganizou a estrutura, o fluxo e a assistência hospitalar para atender à crescente demanda de pacientes críticos, disponibilizando leitos de UTI em diversos setores para atendimento exclusivo aos pacientes acometidos pela forma grave da COVID-19. O período da coleta foi de março de 2020 a abril de 2022.

### População, critérios de inclusão e exclusão

Foram incluídos pacientes adultos (≥ 18 anos), de ambos os sexos, internados na UTI com quadro clínico classificado como grave, definido pela necessidade de suporte intensivo, como ventilação mecânica invasiva (VMI) ou não invasiva, uso de drogas vasoativas e/ou presença de insuficiência respiratória aguda, instabilidade hemodinâmica ou disfunção orgânica^(14)^. O diagnóstico de infecção por SARS-CoV-2 foi confirmado por teste de reação em cadeia da polimerase com transcrição reversa em tempo real em amostra de *swab* nasofaríngeo. Foram incluídos apenas pacientes com registro completo da evolução clínica no prontuário eletrônico institucional. Excluíram-se gestantes, pacientes em cuidados paliativos e casos com dados incompletos ou inconsistentes.

### Protocolo do estudo

A coleta de dados dos prontuários foi realizada de forma retrospectiva e longitudinal por meio de um formulário padronizado e estruturado, elaborado na plataforma eletrônica *Research Electronic Data Capture*. Foi criado um instrumento para rastrear os registros ao longo do tempo. O formulário de dados demográficos e clínicos foi registrado uma única vez, e os resultados dos exames laboratoriais foram registrados na admissão, em 24 horas, em 48 horas e no sétimo dia de internação. Os resultados laboratoriais corresponderam à hemoglobina, leucócitos, plaquetas, creatinina, ureia e d-dímero. O desfecho primário foi o óbito, enquanto o desfecho secundário incluiu a utilização de terapias de suporte, como VMI e diálise. O seguimento teve duração de 30 dias, considerando a admissão na UTI como ponto basal. Os fatores associados (variáveis independentes) ao desfecho primário foram os dados sociodemográficos, as características clínicas e os exames laboratoriais. As variáveis demográficas e clínicas incluíram idade, sexo, comorbidades (hipertensão arterial, arritmias, diabetes mellitus, doenças cardiovasculares, doenças respiratórias, HIV, câncer, doenças hepáticas, obesidade, transplante renal, dislipidemias, alcoolismo, doenças neurológicas e tabagismo) e o uso de medicações na UTI.

### Análise dos resultados e estatística

Para análise de dados, as variáveis quantitativas foram avaliadas quanto à normalidade e homogeneidade por meio do teste de Shapiro-Wilk e teste de Levene, respectivamente. Essas variáveis foram apresentadas por média e desvio padrão (±), quando dentro da normalidade, ou por meio de mediana e intervalo interquartil (IIQ). O tamanho da amostra foi determinado pelo número de pacientes elegíveis no período do estudo, não sendo realizado cálculo amostral prévio por se tratar de amostra por conveniência. As variáveis categóricas foram apresentadas por meio de frequência absoluta e frequência relativa (%). A amostra foi dividida em dois grupos com base no desfecho de sobrevivente e não sobrevivente. Para variáveis de continuidade, foi utilizado o teste U de Mann-Whitney. Para a análise das associações entre variáveis categóricas, utilizou-se o teste qui-quadrado de Pearson (χ2), com correção pelo método de Yates, conforme necessário. A regressão logística com método *stepwise backward* com comparação pelo teste *Likelihood Ratio* foi conduzida para identificar variáveis independentes associadas ao desfecho de óbito. Foram imputadas ao modelo inicial todas as variáveis independentes com p<0,2, realizando-se seis *steps*. Esse método automatizado removeu progressivamente as variáveis menos significativas até a obtenção do modelo final. Para ajuste da multicolinearidade, uma matriz qui-quadrado foi realizada com todas as variáveis independentes do modelo final proposto.

Os estimadores do modelo foram avaliados como *Odds Ratio* (OR). O critério de informação de Akaike foi utilizado como métrica comparativa da qualidade dos modelos estatísticos gerados. Para todas as análises, foi adotado o Intervalo de Confiança de 95% (IC95%), sendo considerado valor de p<0,05 estatisticamente significativo. Para análises significativas, foram utilizados o coeficiente de correlação biserial e o V de Cramer para avaliar a magnitude do tamanho do efeito dos resultados. RStudio^®^ 2023.12.0 e Jamovi^®^ 2.2.5 foram usados para todas as análises.

## RESULTADOS

Foram analisados 715 pacientes com COVID-19, dos quais 506 (70,7%) foram a óbito no hospital (não sobreviventes) e 209 (29,2%) pacientes tiveram alta (sobreviventes). Os dados são apresentados na Tabela 1. A mediana de idade foi de 64 anos (IIQ=55-73), relacionando-se ao desfecho óbito (p<0,01). Notavelmente, os pacientes que não sobreviveram apresentaram uma mediana de nove anos a mais de idade do que os pacientes sobreviventes. A porcentagem do número de pacientes do sexo masculino foi maior no grupo sobrevivente (65,4%).

**Tabela 1:** Características demográficas e basais de pacientes críticos com COVID-19 em Unidade de Terapia Intensiva, São Paulo, São Paulo, Brasil, 2025

Características demográficas e basais	Total (N=715)	Desfecho		Teste estatístico Valor de *p*
		Sobreviventes	Não sobreviventes	
		(n=209)	(n=506)	
**Sexo masculino n(%)**	450 (64,0)	136(65,4)	314 (63,4)	χ^2^_1_=0,24, p=0,62^2^
**Idade Md (IIQ)**	64 (55-73)	57 (47,7,67)	66 (58,75)	**U=34274, p<0,01^1^**
**Raça n(%)**				χ^2^_3_=0,65, p=0,88^2^
Branca	297 (59,2)	120 (58,5)	417 (59,0)	
Negra	60 (12,0)	27 (13,2)	87 (12,3)	
Parda	144 (28,7)	57 (27,8)	201 (28,4)	
Indígena	1 (0,2)	1 (0,5)	2 (0,3)	
**Comorbidades n(%)**	669 (94,2)	189 (91,3)	480 (95,4)	**χ^2^_1_ =4,58, P=0,03^2^**
Hipertensão	453 (67,9)	126 (66,7)	327 (68,4)	χ^2^_1_=0,19, p<0,66^2^
Arritmias	41 (6,2)	8 (4,3)	33 (6,9)	χ^2^_1_=1,67, p<0,20^2^
Diabetes mellitus	272 (40,9)	80 (42,6)	192 (40,3)	χ^2^_1_=0,30, p<0,59^2^
Doença cardiovasculares	162 (24,4)	24 (12,7)	138 (29,1)	**χ^2^_1_ =19,60, p<0,01^2^**
Doença pulmonar obstrutiva crônica e/ou asma	85 (12,7)	21 (11,2)	64 (13,4)	χ^2^_1_=0,58, p<0,45^2^
HIV/AIDS	17 (2,5)	4 (2,1)	13 (2,7)	χ^2^_1_=0,20, p<0,66^2^
Câncer	77 (11,5)	13 (6,9)	64 (13,4)	**χ^2^_1_ =5,62, p<0,02^2^**
Doenças hepáticas	36 (5,4)	2 (1,1)	34 (7,1)	**χ^2^_1_ =9,75, p<0,02^2^**
Obesidade	104 (15,7)	41 (21,7)	63 (13,3)	**χ^2^_1_ =7,27, p<0,01^2^**
Doenças renais	147 (22,1)	33 (17,6)	114 (23,8)	χ^2^_1_=3,11, p<0,08^2^
Dislipidemia	85 (12,8)	33 (17,5)	52 (10,9)	**χ^2^_1_ =5,23, p<0,02^2^**
Alcoolismo	71 (10,7)	15 (8,0)	56 (11,7)	χ^2^_1_=1,92, p<0,17^2^
Doenças neurológicas	59 (8,8)	9 (4,8)	50 (10,5)	**χ^2^_1_ =5,45, P<0,02^2^**
Tabagismo	200 (30,0)	62 (32,8)	138 (28,9)	χ^2^_1_=1,00, p<0,32^2^
**Medicações na UTI n(%)**				
Oseltamivir	87 (12,4)	39 (19,4)	48 (9,6)	**χ^2^_1_ =12,60, p<0,01^2^**
Ceftriaxona	441 (62,3)	125 (61,0)	316 (62,8)	χ^2^_1_=0,21, p<0,65^2^
Claritromicina	165 (23,4)	50 (24,5)	115 (23,0)	χ^2^_1_=0,20, p<0,66^2^
Azitromicina	300 (42,3)	108 (52,7)	192 (38,0)	χ^2^_1_=12,85, p<0,01^2^
Vancomicina	481 (67,7)	81 (39,5)	400 (79,2)	χ^2^_1_=105,15, p<0,01^2^
Piperacilina tazobactam	255 (36,0)	70 (34,3)	185 (36,6)	χ^2^_1_=0,34, p<0,56^2^
Hidroxicloroquina	126 (18,0)	55 (27,2)	71 (14,3)	**χ^2^_1_ =16,28, p<0,01^2^**
Corticosteroides	473 (68,3)	100 (50,3)	373 (75,5)	**χ^2^_1_ =41,76, p<0,01^2^**
Anticoagulantes	631 (91,1)	191 (96,5)	440 (88,9)	**χ^2^_1_ =9,96, p<0,01^2^**
**Medicações de uso crônico**	642 (89,7)			**χ^2^_1_ =9,96, p<0,01^2^**
Não	102(15,9)	44(21,9)	58(13,1)	
Sim	354(55,1)	135(67,2)	219(49,5)	
**Terapia de substituição renal n(%)**	415 (58,2)	61 (29,3)	354 (70,1)	**χ^2^_1_ =100,67, p<0,01^2^**
**VMI n(%)**	574 (80,2)	123 (58,9)	451 (89,0)	**χ^2^_1_ =84,35, p<0,012^2^**

HIV - Vírus da Imunodeficiência Humana; AIDS - Síndrome da Imunodeficiência Adquirida; UTI - Unidade de Terapia Intensiva; VMI - ventilação mecânica invasiva; IIQ - intervalo interquartil; Md - mediana;

2 χ - Pearson;

3 χ - Correção de continuidade de Yates.

Em relação à raça, não houve diferença estatística entre os grupos. As comorbidades foram significativas para o desfecho óbito, em especial as doenças cardiovasculares (29,1%), o câncer (13,4%), a obesidade (13,3%), as dislipidemias (10,9%) e as doenças neurológicas (10,5%), as doenças hepáticas (7,1%) (p<0,05) para todas essas análises. Os pacientes que faziam uso de medicações de uso crônico representaram 55,1% da amostra, e desses que faziam uso regular e contínuo, 67,2% eram do grupo dos sobreviventes.

Pacientes submetidos à VMI e terapia de substituição renal apresentaram altas taxas de mortalidade de 70,1% e 89%, respectivamente (p<0,01). Com relação às medicações para o tratamento de COVID-19 intra-hospitalar nas UTIs, não sobreviveram 49,5% dos pacientes que faziam uso contínuo (p<0,01).

Na Tabela 2, apresentamos os exames laboratoriais e sua associação com o óbito. As plaquetas, a ureia e a creatinina sérica estavam elevadas nas 24 horas de admissão na UTI, com a elevação adicional no sétimo dia após a admissão na UTI (p<0,01).

**Tabela 2:** Teste laboratorial anormal em 24 horas e no sétimo dia da admissão na Unidade de Terapia Intensiva, São Paulo, São Paulo, Brasil, 2025

Exames laboratoriais	Total (N=715)	Desfecho		Valor de *p*
		Sobreviventes	Não sobreviventes	
		(n=209)	(n=506)	
Proteína C reativa				
24 horas	528 (99,8)	150 (99,3)	378 (100,0)	χ^2^_1_=2,51, p=0,28^4^
Sétimo dia	567 (99,8)	167 (99,4)	400 (100,0)	χ^2^_1_=2,39, p=0,29^4^
**Hemoglobina**				
24 horas	364 (51,9)	105 (51,0)	259 (52,2)	χ^2^_1_=0,09, p=0,76^2^
Sétimo dia	41 (6,2)	8 (4,3)	33 (6,9)	χ^2^_1_=0,26, p=0,61^2^
**Hematócrito**				
24 horas	257 (57,4)	99 (55,0)	158 (59,0)	χ^2^_1_=0,69, p=0,41^2^
Sétimo dia	256 (56,3)	94 (52,2)	162 (58,9)	χ^2^_1_=1,98, p=0,16^2^
**Leucócitos**				
24 horas	290 (41,4)	78 (37,9)	212 (42,8)	χ^2^_1_=1,48, p=0,22^2^
Sétimo dia	433 (61,8)	117 (56,8)	433 (61,8)	χ^2^_1_=3,05, p=0,08^2^
**Plaquetas**				
24 horas	228 (32,5)	53 (25,7)	175 (35,3)	**χ^2^_1_=6,06, p=0,01^2^**
Sétimo dia	165 (23,5)	37 (18,0)	128 (25,8)	**χ^2^_1_=4,92, p=0,03^2^**
**Ureia**				
24 horas	432 (62,2)	103 (50,2)	329 (67,1)	**χ^2^_1_=17,55, p<0,01^2^**
Sétimo dia	580 (83,2)	148 (72,2)	432 (87,8)	**χ^2^_1_=25,24, p<0,01^2^**
**Creatinina**				
24 horas	533 (76,8)	140 (68,0)	393 (80,5)	**χ^2^_1_=12,85, p<0,01^2^**
Sétimo dia	613 (87,9)	166 (80,6)	447 (91,0)	**χ^2^_1_=14,97, p=0,01^2^**
**D-dímero**				
24 horas	360 (95,5)	120 (94,5)	240 (96,0)	χ^2^_1_=0,45, p=0,50^2^
Sétimo dia	462 (97,7)	134 (96,4)	328 (98,2)	χ^2^_1_=1,40, p=0,24^2^

2 χ - Pearson;

4 Teste exato de Fisher.

Para o modelo univariável logístico, os resultados mostraram que a categoria etária, o uso de medicamentos na UTI, comorbidades, terapia de substituição renal, VMI e alguns achados laboratoriais foram fatores importantes associados à morte.

A Tabela 3 demonstra que, em relação à idade, indivíduos na faixa etária entre 31-60 anos apresentaram 4,5 vezes mais chances de óbito (OR: 4,51; IC95%: 1,68 - 12,12; p = 0,003) e aqueles entre 61-80 anos tiveram 6,3 vezes mais chances de óbito (OR: 6,30; IC95%: 1,96 - 20,21; p = 0,002), ambos quando comparados aos de faixa etária entre 18-30 anos. O sexo masculino não mostrou associação significativa com óbito (OR: 0,91; IC95%: 0,65 - 1,28; p = 0,623).

**Tabela 3:** Exponencial dos coeficientes estimados segundo o desfecho óbito, *Odds ratio* e Intervalo de Confiança de 95%, São Paulo, São Paulo, Brasil, 2025

Preditor de óbito univariável		*Odds Ratio*	IC95%	Valor de *p*
**Categoria idade (anos)**	**18-30 anos**	**1,50**	(0,56-4,01)	0,416
	**31-60 anos**	**4,51**	**(1,68-12,12)**	**0,003**
	**61-80 anos**	**6,30**	**(1,96-20,21)**	**0,002**
**Sexo**	**Masculino**	**0,91**	(0,65-1,28)	0,623
**Medicações na UTI**	Oseltamivir	**0,44**	**(0,27-0,70)**	**<0,001**
	Ceftriaxona	1,08	(0,77-1,51)	0,646
	Claritromicina	0,91	(0,62-1,34)	0,658
	Azitromicina	**1,81**	**(1,30-2,51)**	**<0,001**
	Vancomicina	**5,83**	**(4,09-8,30)**	**<0,001**
	Piperacilina tazobactam	1,10	(0,78-1,55)	0,56
	Hidroxicloroquina	**0,44**	**(2,99-0,66)**	**<0,001**
	Corticosteroides	3,05	(2,16-4,31)	**<0,001**
	Anticoagulantes	**0,29**	**(0,13-0,65)**	**0,003**
**Comorbidades**		1,98	(1,04-3,76)	0,035
	Hipertensão	1,08	(0,75-1,55)	0,664
	Arritmias	1,67	(0,75-3,69)	0,201
	Diabetes mellitus	0,90	(0,64-1,28)	0,587
	Doenças cardiovasculares	2,81	(1,75-4,51)	**<0,001**
	Doença pulmonar obstrutiva crônica e/ou asma	1,22	(0,72-2,07)	0,446
	HIV/AIDS	1,29	(0,41-4,00)	0,659
	Câncer	**2,09**	**(1,12-3,89)**	**0,02**
	Doenças hepáticas	**7,17**	**(1,70-30,17)**	**0,007**
	Obesidade	**0,55**	**(0,35-0,85)**	**0,008**
	Transplante renal	1,47	(0,95-2,26)	0,079
	Dislipidemia	0,57	(0,36-0,92)	0,023
	Alcoolismo	1,52	(0,83-2,76)	0,168
	Doenças neurológicas	**2,33**	**(1,12-4,85)**	**0,023**
	Tabagismo	0,83	(0,57-1,19)	0,318
**Terapia de substituição renal**		**5,65**	**(3,96-8,05)**	**<0,001**
**VMI**		**5,61**	**(3,79-8,30)**	**<0,001**
**Achados laboratoriais**	Hemoglobina após 24 horas	1,05	(0,75-1,45)	0,301
	Hemoglobina no sétimo dia	1,08	(0,78-1,50)	0,608
			
	Hematócrito após 24 horas	1,17	(0,80-1,72)	0,407
	Hematócrito no sétimo dia	1,31	(0,89-1,91)	0,160
			
	Linfócitos após 24 horas	1,22	(0,88-1,71)	0,224
	Linfócitos no sétimo dia	1,34	(0,96-1,87)	0,081
			
	Plaquetas após 24 horas	1,57	(1,09-2,26)	**0,014**
	Plaquetas no sétimo dia	1,58	(1,05-2,38)	**0,027**
			
	Ureia após 24 horas	2,02	(1,45-2,82)	**<0,001**
	Ureia no sétimo dia	2,77	(1,84-4,16)	**<0,001**
			
	Creatinina após 24 horas	1,95	(1,34-2,81)	**<0,001**
	Creatinina no sétimo dia	2,44	(1,54-3,89)	**<0,001**
			
	D-dímero após 24 horas	1,40	(0,52-3,76)	0,506
	D-dímero no sétimo dia	2,04	(0,61-6,79)	0,246

*HIV - Vírus da Imunodeficiência Humana; AIDS - Síndrome da Imunodeficiência Adquirida; UTI - Unidade de Terapia Intensiva; VMI - ventilação mecânica invasiva; IC95% - Intervalo de Confiança de 95%.*

Quanto às comorbidades, a presença de qualquer comorbidade aumentou as chances de óbito em 98% (OR: 1,98; IC95%: 1,04 - 3,76; p = 0,035), com destaque para doenças cardiovasculares (OR: 2,81; IC95%: 1,75 - 4,51; p < 0,001), câncer (OR: 2,09; IC95%: 1,12 - 3,89; p = 0,02), doenças hepáticas (OR: 7,17; IC95%: 1,70-30,17; p=0,007), obesidade (OR: 0,55; IC95%: 0,35-0,85; p=0,008) e doenças neurológicas (OR=2,33; IC95%: 1,12-4,85; p=0,023). Portanto, comorbidades como doenças cardiovasculares, câncer, doenças hepáticas, obesidade, dislipidemias e doenças neurológicas foram as condições crônicas significativas para o desfecho do óbito (p<0,01). Vale ressaltar que, quando o paciente era submetido a terapias de suporte, como VMI ou terapia de substituição renal, suas chances aumentaram em mais de 5,6 vezes de ir a óbito para ambos: OR: 5,61; IC95%: 3,96-8,05; p<0,001 e OR:5,65, IC95%: 3,79-8,30; p<0,001, respectivamente.

Por outro lado, conforme os achados da terapia renal substitutiva, os biomarcadores de função renal, como ureia e creatinina sérica, foram preditores de óbito com mais de duas vezes o risco de morte no sétimo dia, e as plaquetas nas 24 horas e no sétimo dia demonstraram relevância nessa casuística, atingindo, depois de sete dias (OR:1,58; IC95%:1,05-2,38; p=0,027).

Para o modelo final da regressão pelo método *stepwise*, modo *backward* (Tabela 4), obtivemos as variáveis selecionadas com p menor que 0,2 e foram realizados seis steps com o modelo final. As análises multivariadas foram então realizadas. Após o ajuste, 18 variáveis foram incluídas no modelo final, e a interação vancomicina + VMI, quando adicionada ao modelo para fins de controle, apresentou melhores ajustes dos dados e diferença significativa em relação ao modelo final.

**Tabela 4:** Modelo final da regressão multivariada do tipo *stepwise backward* com e sem ajuste para interação, *Odds Ratio* e Intervalo de Confiança de 95% para os fatores associados ao desfecho óbito, São Paulo, São Paulo, Brasil, 2025

Variáveis	Modelo não ajustado			Modelo ajustado		
	*Odds Ratio*	IC95%	Valor de *p*	*Odds Ratio*	IC95%	Valor de *p*
18-30 anos	0,033	(0,006-0,164)	<0,001	0,03	(0,006-0,158)	<0,001
31-60 anos	0,108	(0,034-0,299)	<0,001	0,09	(0,026-0,273)	<0,001
61-80 anos	0,382	(0,124-1,047)	0,076	0,35	(0,103-1,044)	0,078
VMI	5,965	(3,251-11,21)	<0,001	28,5	(9,502-103,7)	<0,001
Admissão na UTI	0,198	(0,028-0,828)	0,051	0,24	(0,034-1,010)	0,087
Vancomicina	3,705	(2,217-6,258)	<0,001	29,6	(8,459-122,1)	<0,001
Corticosteroides	1,889	(1,129-3,164)	0,015	1,83	(1,090-3,100)	0,022
Anticoagulantes	0,170	(0,047-0,506)	0,003	0,16	(0,040-0,512)	0,005
Doenças cardiovasculares	4,599	(2,344-9,521)	<0,001	4,82	(2,375-10,47)	<0,001
Câncer	2,560	(1,135-6,228)	0,030	2,31	(1,012-5,723)	0,056
Doença hepática	10,19	(2,392-72,10)	0,005	10,11	(2,380-71,15)	0,005
Obesidade	0,584	(0,315-1,087)	0,088	0,590	(0,319-1,101)	0,094
Dislipidemia	0,580	(0,282-1,199)	0,139	0,578	(0,279-1,213)	0,142
Doenças neurológicas	2,474	(0,880-7,966	0,104	2,635	(0,892-9,098)	0,099
Terapia renal substitutiva	6,135	(3,637-10,59)	<0,001	6,278	(3,683-10,96)	<0,001
Plaquetas após 24 horas	1,963	(1,036-3,741)	0,039	1,927	(1,011-3,693)	0,046
Plaquetas no sétimo dia	0,505	(0,239-1,041)	0,068	0,507	(0,235-1,062)	0,076
Ureia após 24 horas	0,663	(0,398-1,105)	0,114	0,667	(0,397-1,121)	0,125
Ventilação ^*^Vancomicina	-	-	-	0,077	(0,017-0,303	<0,001

UTI - Unidade de Terapia Intensiva; VMI - ventilação mecânica invasiva; IC95% - Intervalo de Confiança de 95%; Ventilação^*^Vancomina- Interação Ventilação +Vancomicina.

## DISCUSSÃO

Os pacientes não sobreviventes corresponderam a uma alta e alarmante taxa de mortalidade. Esses achados reforçam a gravidade da COVID-19, especialmente em contextos de UTI. A análise de dados revelou que a idade avançada e a presença de comorbidades foram os principais fatores para desfechos fatais e para o uso de terapias de suporte, como diálise e ventilação mecânica, enquanto os biomarcadores das funções renal, hematológica e hepática se destacaram como indicadores críticos da gravidade do quadro clínico dos pacientes.

O alto índice de mortalidade do nosso estudo foi discrepante em relação a prévios estudos, que revelaram índices menores variando de 21,7% a 47,3% de pacientes hospitalizados no Brasil. Esses estudos, entretanto, são originados do SIVEP-Gripe, e podemos levar em consideração que a variação dessas taxas ocorreu devido a diferentes disponibilidades de acessos a tecnologias de saúde nas diferentes regiões do país^(6,15)^.

A idade avançada foi determinante para o número elevado de pacientes não sobreviventes nesta investigação. Quando categorizamos os pacientes por faixa etária, observamos que suas chances de óbito aumentavam em comparação aos mais jovens. Os idosos apresentam um estado pró-inflamatório de baixo grau que, juntamente com as peculiaridades da imunossenescência e o processo de envelhecimento imunitário, pode favorecer a síndrome da tempestade de citocinas e o aumento de mediadores inflamatórios séricos^(16)^.

Este estudo não encontrou significância estatística na relação entre a raça e os casos de mortalidade. Por outro lado, o estudo de Baqui *et al*.^(17)^ encontrou dois efeitos distintos, com um aumento da mortalidade na região Norte (efeito regional) e na população parda (efeito etnia), sendo especulado que o efeito étnico pode estar relacionado ao acesso aos cuidados de saúde; portanto, nossa análise apoia essa justificativa, pois a região Sudeste concentrou, durante a pandemia, a maior parte dos recursos físicos, humanos e tecnológicos de saúde do Brasil.

A presença de comorbidades foi significativamente associada ao óbito (p=0,032), sendo que 91,3% dos sobreviventes também apresentavam alguma condição pré-existente. Comorbidades podem predispor a uma resposta imune disfuncional, impactando negativamente a evolução da COVID-19^(18)^. Doenças cardiovasculares, em particular, estão fortemente ligadas à gravidade da doença, podendo resultar em miocardite, síndromes coronárias agudas ou insuficiência cardíaca, com risco de mortalidade até cinco vezes maior em comparação a pacientes sem doenças cardiovasculares^(19)^. Neste estudo, doenças cardiovasculares aumentaram em 2,81 vezes a chance de óbito, em concordância com achados de Almeida-Pititto^(20)^.

As doenças hepáticas aparecem como um importante fator envolvendo o grupo óbito, com mais de sete vezes a chance de pior prognóstico, corroborando dados que mostram que a doença hepática crônica apresentou chances significativamente maiores de desenvolver uma forma grave da doença^(21)^. Um perfil pró-inflamatório dependente de COVID-19 e uma supressão imunológica promovem o microambiente ideal para iniciação e evasão imunológica de células malignas, progressão tumoral e metástases, contribuindo para a progressão de vários tipos de câncer e mortalidade pela SARS-CoV-2^(22)^.

Além dessas comorbidades, neste estudo brasileiro, a caracterização do perfil também encontrou maior prevalência nas doenças neurológicas. O cuidado de pacientes com COVID-19 na UTI, especialmente daqueles que apresentam acidente vascular cerebral, é descrito como associado a altas taxas de mortalidade. Portanto, as comorbidades neurológicas merecem maior atenção no direcionamento dessa população para uma triagem viral proativa^(23)^.

Esses dados permitiram rever as principais comorbidades pré-existentes e como o sistema público de saúde brasileiro deve priorizar esses grupos e manter as campanhas de vacinação, evitando a lacuna que ocorreu em relação à compreensão do valor preventivo da vacina e a hesitação em seu uso, uma vez que ela protege não apenas os indivíduos vulneráveis, mas também o desenvolvimento de doenças graves e óbitos por COVID-19^(24)^. Na admissão, 80,5% dos pacientes apresentavam evidências de lesão renal aguda, avaliadas por creatinina sérica, exame rotineiro na UTI. Estudos anteriores relatam valores normais de creatinina, sódio e potássio, destacando a importância do acompanhamento longitudinal desde a admissão.

Apesar de o presente estudo não ter estratificado o tipo de lesão renal, essa elevação da creatinina tem sido amplamente reportada como preditor de morbimortalidade. Dessa forma, a atenção voltada à coleta diária de creatinina na prática clínica concretiza a creatinina como um biomarcador útil para aludir atenção especial ao paciente renal com COVID-19. Outro marcador significante foi a ureia, corroborando um marcador das características da tempestade inflamatória e gravidade da doença^(25)^. Além disso, o início da terapia representou 70,1% do grupo óbito, valor esse discrepante quando comparado aos dados dos Estados Unidos da América, em que 20,6% dos pacientes necessitaram de terapia renal substitutiva em 14 dias de UTI. Esses dados expressivos podem estar relacionados ao mecanismo de dano direto do vírus mediado pela coexpressão e atividade da enzima conversora de angiotensina 2, mecanismos importantes que conduzem a uma lesão renal em pacientes com infecção mais crítica^(26)^.

Muitos estudos emergem acerca dos aspectos da associação da VMI e dos desfechos desses pacientes, podendo ser multifatorial. No nosso estudo, a mortalidade foi de 89%, o que é particularmente elevado, similar a uma coorte que, aos 28 dias, apresentou 81,1% de mortalidade^(27)^. Esses dados sugerem a vulnerabilidade de pacientes em VMI a lesões pulmonares induzidas pelo ventilador, colocando em pauta fatores que ainda são amplamente discutidos quando consideramos estratégias para intubação. Não obstante, quando analisamos a interação da covariável VMI e vancomicina após ajuste do modelo, poderíamos apontar uma outra discussão sobre o aumento do uso de antibióticos associado ao uso da VMI nos casos graves e infecções secundárias, desencadeando a utilização de antibióticos de amplo espectro de forma indiscriminada^(28)^.

### Limitações do estudo

O estudo apresentou delineamento retrospectivo e único centro baseado em dados secundários de prontuários para fins clínicos. O estudo abrangeu a fase inicial da pandemia, incluindo a primeira e segunda ondas da COVID-19, caracterizadas por alta incidência e hospitalização, sem considerar o impacto das diferentes variantes ou da vacinação, o que pode limitar a generalização dos resultados para fases posteriores da doença. Embora o delineamento seja de coorte, optou-se por regressão logística para identificar fatores independentes associados ao óbito, ajustando múltiplos preditores simultaneamente. Reconhece-se que, devido à alta frequência do desfecho, os OR podem superestimar a magnitude das associações em comparação ao risco relativo, devendo essa limitação ser considerada na interpretação dos resultados; entretanto, a regressão logística continua adequada para a seleção de variáveis independentes e construção de modelos explicativos.

### Contribuições para a área da enfermagem, saúde ou políticas públicas

Este estudo permitiu associar e analisar o desfecho óbito devido à COVID-19 no período do início da pandemia e caracterizar minunciosamente essa população, demonstrando a importância de promover a prevenção e controle dos fatores de risco em grupos mais vulneráveis à gravidade. Não obstante, o estudo tem contribuições importantes, demonstradas no modelo de regressão logística para predizer mortalidade, que podem vir a ser semelhante em outras situações epidemiológicas semelhantes.

## CONCLUSÕES

A idade avançada e comorbidades crônicas, como doenças cardiovasculares, doenças hepáticas, câncer, diálise, VMI e contextos clínicos de gravidades e coinfecções, demonstraram ser os fatores associados para predizer a mortalidade dos pacientes graves com COVID-19. O modelo multivariado apresentou boa capacidade discriminatória, identificando fatores independentes associados ao óbito em pacientes graves com COVID-19.

## Data Availability

Os dados de pesquisa estão disponíveis em repositório: https://doi.org/10.48331/SCIELODATA.EULYWL.
